# Colony Failure Linked to Low Sperm Viability in Honey Bee (*Apis mellifera*) Queens and an Exploration of Potential Causative Factors

**DOI:** 10.1371/journal.pone.0147220

**Published:** 2016-02-10

**Authors:** Jeffery S. Pettis, Nathan Rice, Katie Joselow, Dennis vanEngelsdorp, Veeranan Chaimanee

**Affiliations:** 1 Bee Research Laboratory, USDA-ARS, Beltsville, Maryland, United States of America; 2 Entomology Department, University of Maryland, College Park, Maryland, United States of America; 3 Department of Biotechnology, Maejo University Phrae Campus, Rong Kwang, Phrae, Thailand; San Diego, UNITED STATES

## Abstract

Queen health is closely linked to colony performance in honey bees as a single queen is normally responsible for all egg laying and brood production within the colony. In the U. S. in recent years, queens have been failing at a high rate; with 50% or greater of queens replaced in colonies within 6 months when historically a queen might live one to two years. This high rate of queen failure coincides with the high mortality rates of colonies in the US, some years with >50% of colonies dying. In the current study, surveys of sperm viability in US queens were made to determine if sperm viability plays a role in queen or colony failure. Wide variation was observed in sperm viability from four sets of queens removed from colonies that beekeepers rated as in good health (n = 12; average viability = 92%), were replacing as part of normal management (n = 28; 57%), or where rated as failing (n = 18 and 19; 54% and 55%). Two additional paired set of queens showed a statistically significant difference in viability between colonies rated by the beekeeper as failing or in good health from the same apiaries. Queens removed from colonies rated in good health averaged high viability (ca. 85%) while those rated as failing or in poor health had significantly lower viability (ca. 50%). Thus low sperm viability was indicative of, or linked to, colony performance. To explore the source of low sperm viability, six commercial queen breeders were surveyed and wide variation in viability (range 60–90%) was documented between breeders. This variability could originate from the drones the queens mate with or temperature extremes that queens are exposed to during shipment. The role of shipping temperature as a possible explanation for low sperm viability was explored. We documented that during shipment queens are exposed to temperature spikes (<8 and > 40°C) and these spikes can kill 50% or more of the sperm stored in queen spermathecae in live queens. Clearly low sperm viability is linked to colony performance and laboratory and field data provide evidence that temperature extremes are a potential causative factor.

## Introduction

Honey bees, *Apis mellifera* live in highly eusocial colonies that normally contain a single queen. With colony success vested highly in this one individual, her health is of utmost importance to colony growth and survival [1]. Any decline in queen health can have an adverse effects in colony performance if a colony fails to supersede (replace) the failing queen[2,3]. Queens are being replaced at a very high rate in the U.S. [4] compared to historic norms and little is known about the putative causes of these high failure rates [5].

In the US honey bee colonies have been dying at an unacceptably high rate over the past years [6–8]. These losses, at least in part, are thought to be the result of queen failures, as 50% or more of queens are replaced within 6 months in some commercial operations [4, 9]. This is compares to historic data where queens lived 2–3 years [2, 10].

Honey bee colonies are susceptible to a variety of pests and diseases. Beekeepers rely on pesticides to control parasitic mites and antibiotics to control certain diseases. These products can impact colony health [11–15]. Specifically, miticides used to control Varroa mites accumulate in wax comb and can impact drone, [16, 17] queen [18–21] and colony survival [9, 22].

There are several reasons queens can fail, including poor mating, pathogen infection [23–26] and drones can transmit viruses to queens via semen [27]. However, these biotic factors seem an unlikely explanation for reported high failure rates as a survey of commercial queens in 2007 showed that queens were well mated (sperm number 4 million) with an average of 16 drones and had low disease incidence [5]. Little work has been done on the role of abiotic factors, such as temperature and pesticide exposure on queen, specifically her stored sperm, health. So why are beekeepers having high queen failures if the queens’ disease levels are low? The rearing of queens is the same as it has been for 100 years or more [3, 28] and little attention has been given to the actual process of rearing better queens [29, 30]. Much attention has been focused on genetics [5, 31–36] but queen shipping conditions have been largely ignored.

To investigate possible reasons for the high rate of queen failures in the U.S., three sets of data were collected; 1) beekeepers were asked to send live queens from colonies that were, in their opinion, in good or failing health within the same apiaries, 2) the queens from six commercial queen breeders were shipped to allow for the monitoring temperatures experienced by queens during shipment and to determine background pathogen levels in U.S. queens sold commercially, 3) laboratory experiments were performed to explore the possible role of temperature extremes on sperm viability in mated queens.

## Materials and Methods

We obtained honey bee queens (*Apis mellifera L*.) from two sources, buying them from queen breeders or by removing them from colonies owned by commercial beekeepers or USDA-ARS. The purchased queens originated from six U. S. queen breeders and were ca. 15–40 days of age; queens removed from colonies were of unknown age. Newly mated queens from queen breeders, were shipped alive in screened cardboard boxes with loose attendant bees surrounding the queens that were held in individual cages within the battery box as it is known. Older queens removed from commercial colonies were shipped in small wooden queen cages with 4–6 attendant worker bees per cage (known as Benton cages). Sperm viability assessments were made following the methods of Collins and Donoghue [37]. Briefly, queens were dissected alive by removing the head and then the abdomen opened to remove the spermathecae from queens. Spermathecae were placed in a 1.5ml eppendorph tubes containing 20 microliters of buffer D [37]. Each spermatheca was pierced with a needle and gently swirled in the buffer to release the sperm. The bulk of the spermathecal wall was then removed and dyes added that differentially stained live and dead sperm. From the dyed sample, three aliquots of 4ml each were placed on a glass microscope slide and using a florescent light source on a compound microscope (Axioskop 2 plus, Carl Zeiss at 400x), live-dead determination were made. One hundred sperm were scored as live or dead based on color by scanning the field of view, moving to new areas in a random pattern and scoring the first 100 sperm encountered. This was repeated on two additional independent aliquots, the high and low values thrown out and the middle value retained as a measure of sperm viability for an individual queen.

### Queens from Commercial Beehives

Beekeepers who had complained of queen failures were asked to rate a set of honey bee colonies in a single apiary as being in good or poor health and to send live queens from those colonies for analysis at USDA-ARS Beltsville, MD. The first two sets of queens (n = 18 & 19) received were all from failing colonies, as beekeepers were reluctant to remove queens from colonies in good health. In Beltsville a set of 12 queens from research colonies in apparent good health were sacrificed to serve as a comparative group for the commercial queens sent from the two commercial beekeepers. A third set of queens from commercial colonies were obtained when a beekeeper who replaces queens on a yearly basis, sent 28 older queens but without hive ratings. Upon making a second request, two beekeepers sent paired sets of queens (one set from the east and western U.S. respectively) that contained queens from healthy and failing colonies as rated by the beekeeper. Queen cages were marked with the colony ratings by the beekeeper but these codes were kept blind from the individual performing the sperm viability determinations as a separate individual dissected the queens and recorded colony condition.

The software JMP version 11.0 for Windows (SAS Institute Inc.) was used for statistical analysis. Comparisons between sperm viability values for the first four sets of queens were made using ANOVA. Viability values for the paired queens from the same apiaries (healthy vs. failing colonies) from the east and west coast commercial beekeepers were made using a paired t-test. Differences were considered statistically significant when alpha was < 0.05.

### Queen Breeder Survey, Shipping Temperatures and Pathogens

Mated queens in paired sets were shipped in July 2014 via US Postal Service (USPS, n = 10) or United Parcel Service (UPS, n = 10) from each of six queen breeders to Beltsville, MD to examine shipping temperatures queens might experience and to survey for background levels of three common pathogens in commercial queens. Each shipment contained two thermocouples (Sentry Inc.) set to record at 10 minute intervals. Upon arrival at USDA-ARS Beltsville bee lab, queens were sacrificed and live-dead sperm determinations made. Additionally, abdomens of 12 queens per breeder (6 from each of the 2 shipments) were analyzed individually for virus (BQCV and DWV) and *Nosema ceranae* levels using established methods [38]. Temperature probes were recovered and data compared between the two probes to verify accuracy and functionality and only the data from one probe used for analysis. To examine consistency of queens by breeder, a second shipment of 10 queens per breeder were obtained in September 2014 from five of the same breeders that shipped queens in July. Differences in viability between shipment methods was compared using a paired t-test. Potential differences in viability between queen breeders was compared separately for the July and September shipments using a Steel-Dwass non-parametric test (JMP version 11.0 SAS Institute Inc.)

### Laboratory queen exposures to temperature extremes

Laboratory studies were conducted to explore the role of temperature extremes on sperm viability. A set of 60 queens were obtained via United Parcel Service from a single queen breeder and randomly sub-divided and subjected to either 4°C for one, two, or four hours or 40°C for one or four hours with control queens held at 30°C. Following temperature exposures, all queens were held an additional six days at 30°C to allow time for any detrimental effects to on sperm to be realized. On day seven all queens were sacrificed and sperm viability measured. Comparisons in viability at different time intervals and temperatures was made using a Steel-Dwass test and Tukey HSD test to separate means when significance was found.

## Results

### Queens from Commercial Colonies

**Fig 1:**
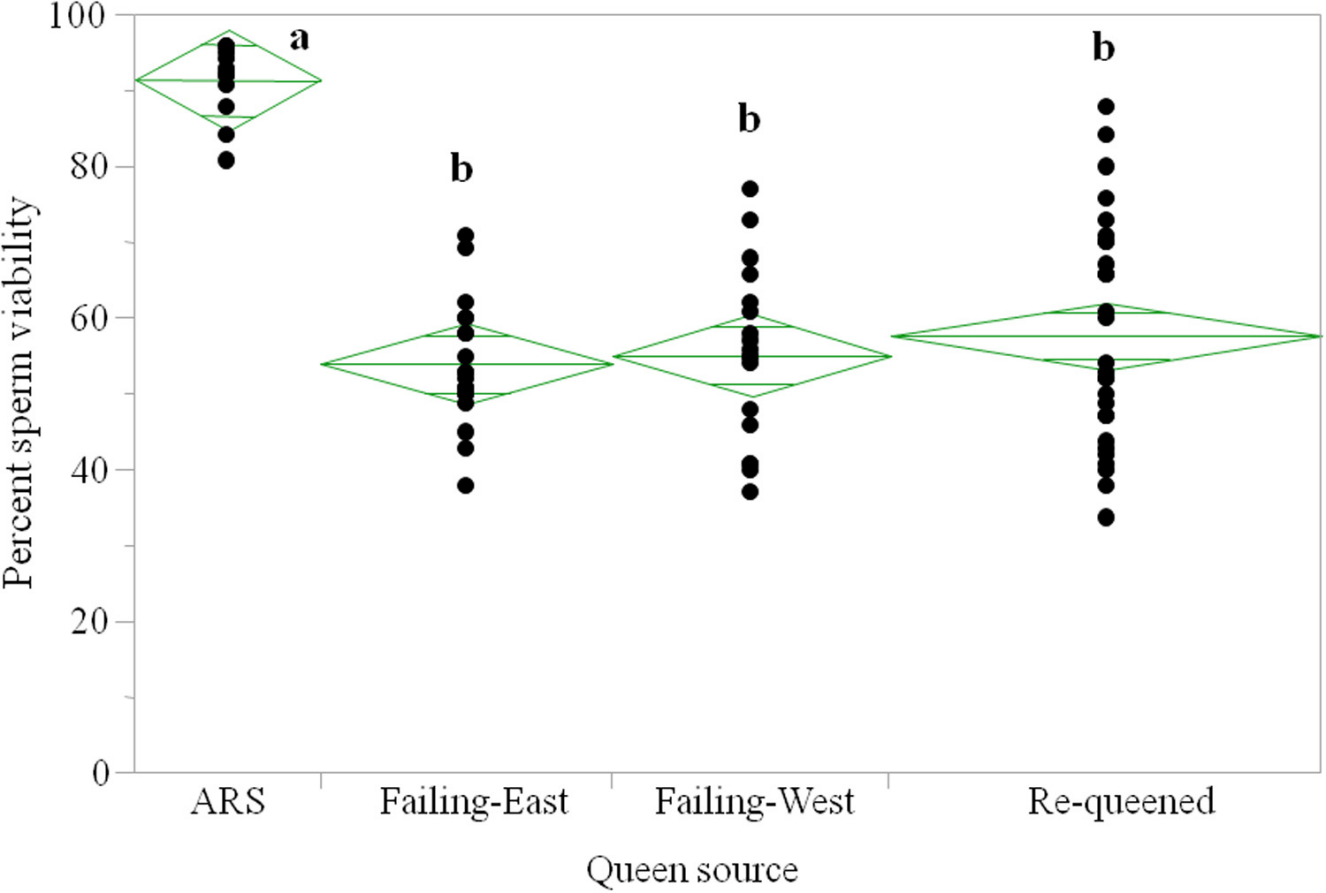
Sperm viability in honey bee queens removed from four different geographic locations; colonies managed by USDA-ARS in Maryland were in apparent good health (n = 12 queens), two commercial beekeeping operations where the beekeeper rated the colonies as in failing health (western U.S. n = 18, eastern U.S. n = 19) and a commercial beekeeper in California who removed queens during routine queen replacement without reguard to colony health (requeen n = 28) where most queens were considered to be ca. one year in age. The actual age of all queens is unknown. Letters indicate significant differences between means by queen source (ANOVA, P = 0.0001).

**Fig 2:**
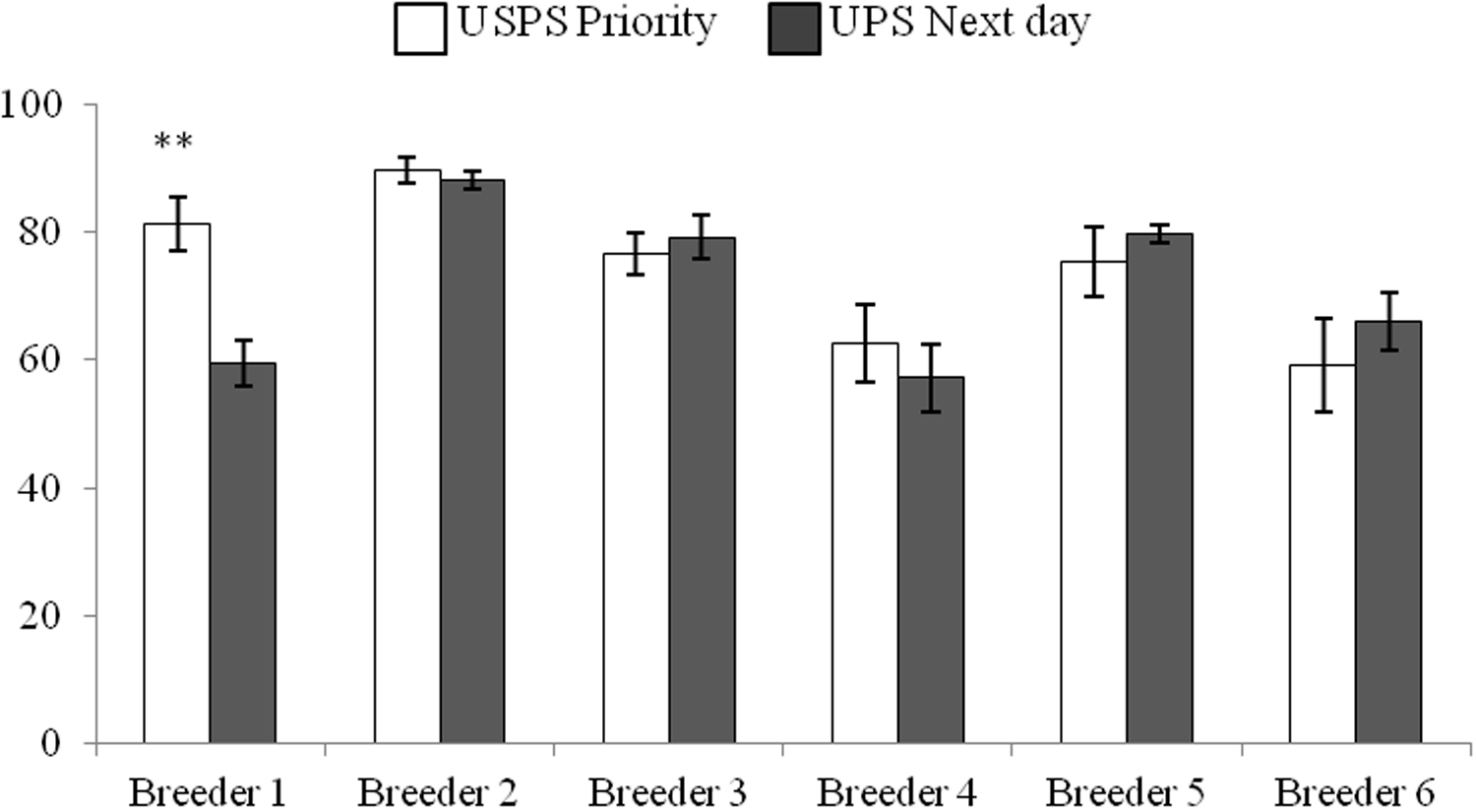
Sperm viability in queens heading colonies that were rated as in healthy or failing health. Queens are from two commercial beekeeping operations, colony heath was rated by the beekeeper and sperm viability assessments conducted blind relative to hive ratings. Data are from queens removed from a single apiary in either the east coast (healthy n = 8 and failing n = 14) or west coast (healthy n = 9 and failing n = 12) and asterisk indicate significant differences in viability within apiaries (paired t-test P<0.05).

The first two sets of queens from colonies in failing health had low sperm viability (Fig 1) with the queens removed from colonies on the west coast averaging 55% viable sperm while east coast queens averaged 54% viability. The queens that were removed during colony re-queening averaged 57% sperm viability. By comparison, the 12 queens removed from colonies in Beltsville, MD at the same time of year (July) as the commercial queens were obtained, averaged 92% sperm viability which was significantly higher than the other three groups of queens (ANOVA, p = 0.001). The paired queens from colonies rated as failing or in good health had significant differences in viability (paired t-test, P<0.05) from both the east and west coast beekeepers, with low sperm viability in queens heading failing colonies (Fig 2).

### Queen Breeder Survey, Shipping Temperatures and Pathogens

**Fig 3:**
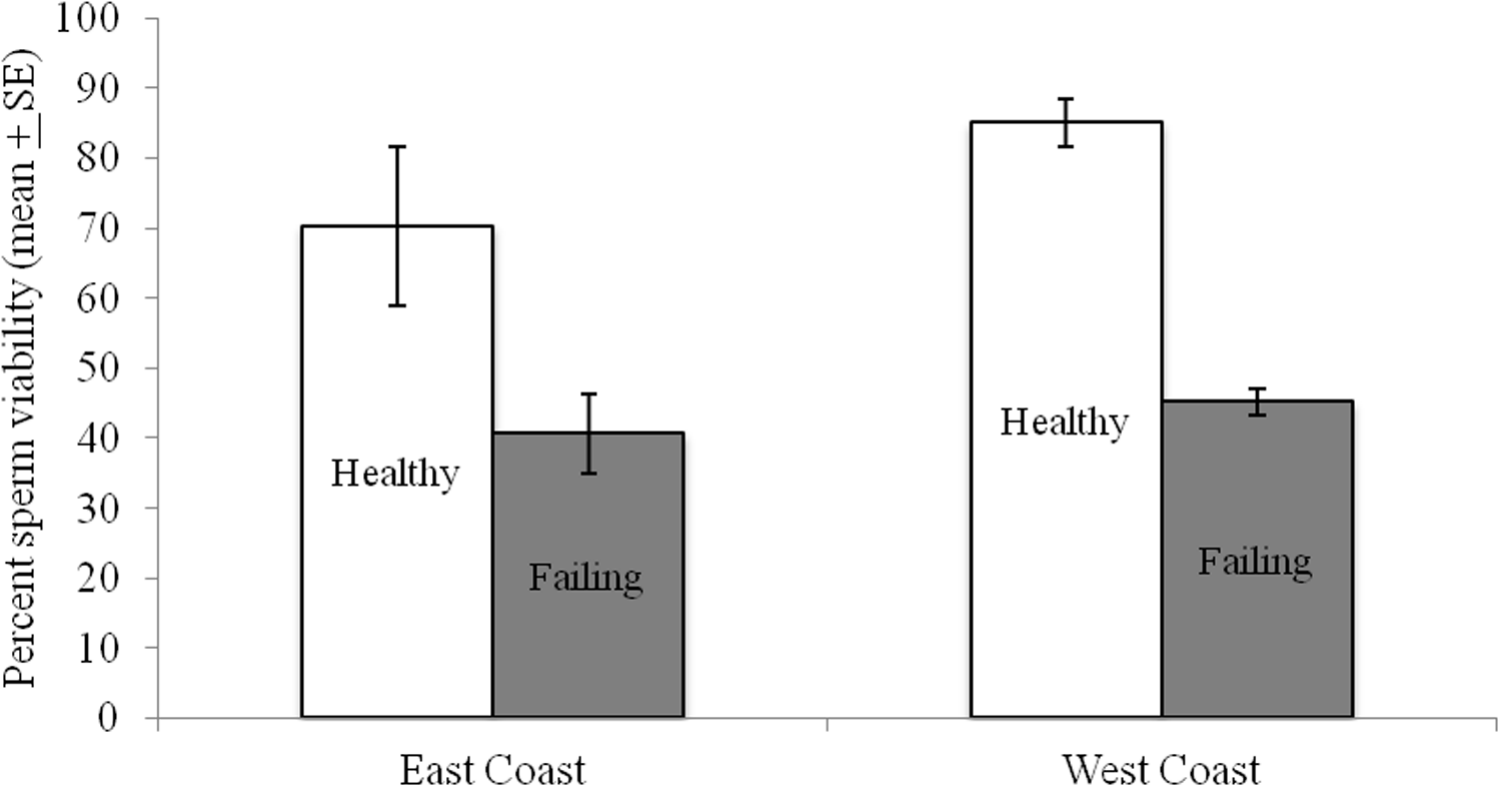
Percent sperm viability in queens (n = 10 per shipping method / breeder) obtained from six queen breeders across the U.S. utilizing two shipping methods, US Postal Service Priority (USPS) and United Parcel Service (UPS). Queen shipments contained temperature monitors and significant difference in viability by shipping method from Breeder #1 represent a cold spike where the queens were exposed to 8°C for two hours. ** indicated significant differences (p<0.01, paired t-test).

**Fig 4:**
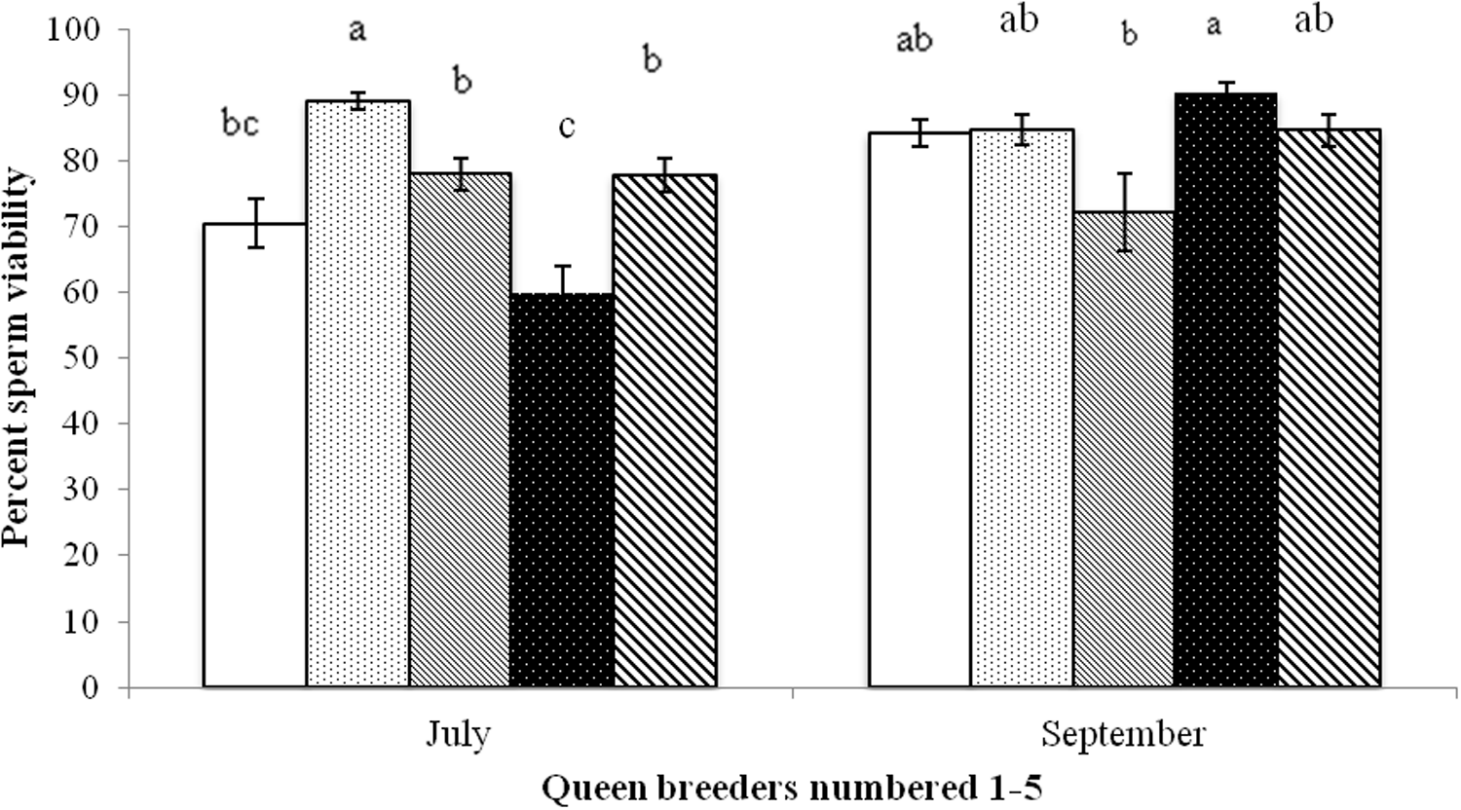
Percent sperm viability in queens (n = 10 per breeder) obtained in July and September from each of five queen breeders in the states of Georgia, California and Hawaii in the U.S. Letters over bars indicate significant differences (p<0.05 Steel-Dwass test).

**Fig 5:**
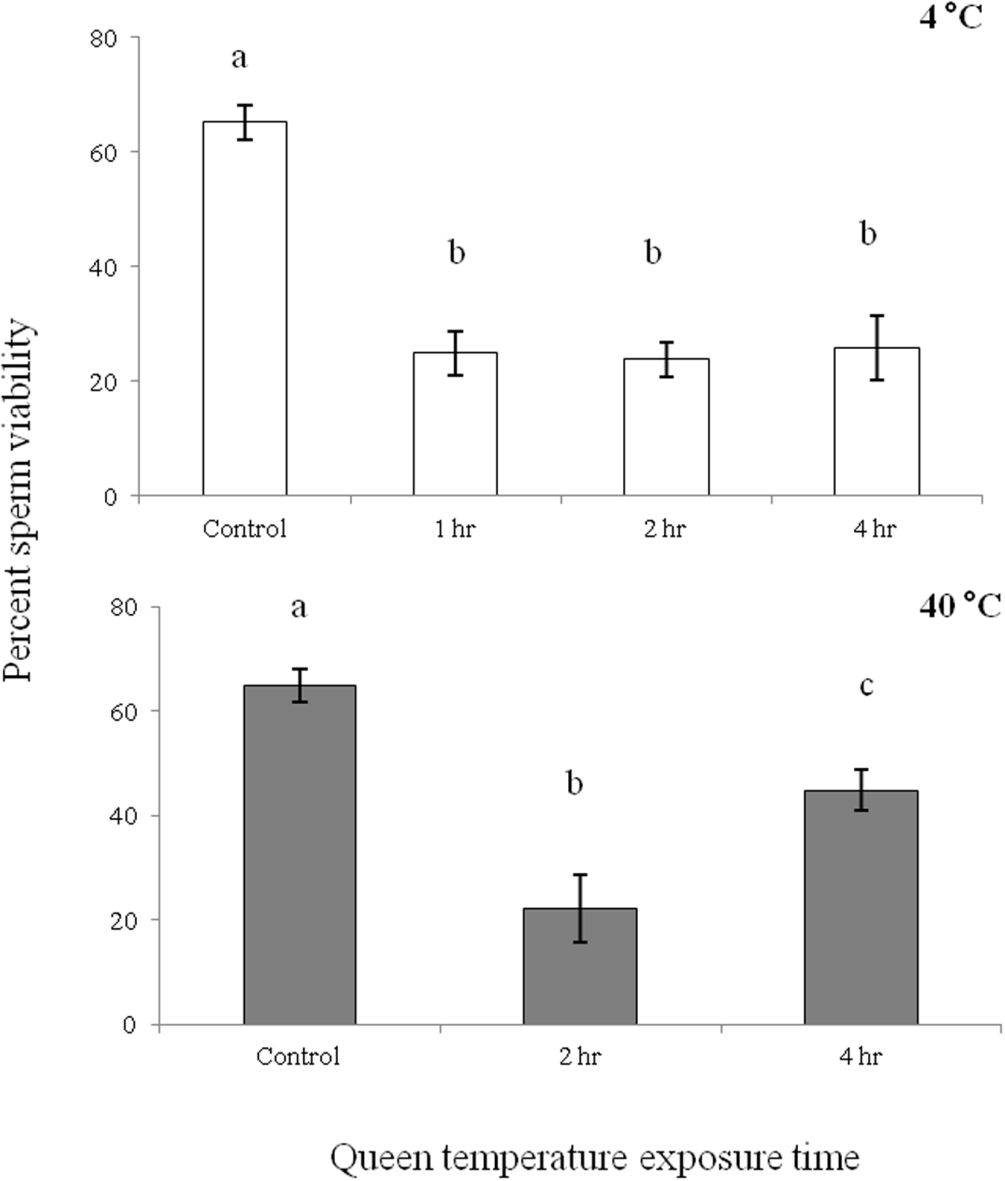
Sperm viability from 60 honey bee queens (n = 10 / temperature and time interval) exposed to high (40°C) and low (4°C) temperatures for varying lengths of time. All queens were from a single source and control queens were held at 30°C for the 7 day test period, with queens exposed to experimental temperatures on day one. Letters over bars indicate significant differences (0<0.001 for all comparisons, 4°C = Steel-Dwass Method, 40°C = ANOVA Tukey-Kramer HSD).

Sperm viability values varied by breeder, with one breeder producing queens with noticeably higher viability (ca. 90%) than the other five (Fig 3). Breeders four and five had values below a proposed acceptable level of 80%. The two shipping methods differed significantly in only one case with breeders one (p<0.01, paired t-test). The temperature probes from breeder one in the shipment with lower viability (USPS) recorded a low spike in temperature of 8°C for two hours. All other shipments showed temperature values within an acceptable range of 15–35°C. Significant differences were observed when the breeders were separately compared in July and September (p<0.05 Steel-Dwass test, Fig 4). The laboratory exposure of queens to extreme temperatures resulted in significant reductions in viability after only one or two hours at either high or low temperatures (p<0.05 Steel-Dwass test, Fig 5). Exposures longer than 1–2 hours at 4°C or 40°C did not result in additional increased mortality.

The pathogen levels in queens from the six breeders are given in Table 1, with all three pathogens present in queen stocks from each breeder with the exception of BQCV being absent from the queens from breeder five. In general, queens had high prevalence of DWV followed by *Nosema ceranae* and low prevalence of BQCV.

**Table 1:** Percentage of honey bee queens infected with Deformed Wing Virus (DWV), Black Queen Cell Virus (BQCV) and *Nosema ceranae* (n = 12 queens per breeder, July 2013) from six commercial queen breeders in the US as detected by rtPCR.

	Percentage of queens infected (n = 12)		
	DWV	BQCV	*Nosema ceranae*
1	75	8	67
2	67	8	42
3	100	25	92
4	75	8	25
5	42	0	67
6	83	17	42

## Discussion

Honey bee colonies that were rated as failing by beekeepers were headed by queens that had low sperm viability compared to queens heading colonies in apparent good health. This relationship between low sperm viability and poor colony performance was most striking in queens removed from colonies within the same apiary owned by two commercial beekeepers. Sperm viability was low in queens removed during routine re-queening from a separate commercial beekeeper when compared to the queens at the USDA-ARS laboratory and previous reports on queen health [39, 40]. A survey of newly-mated commercially available queens from breeders across the U.S. showed wide variation in queen quality based on both sperm viability values and on virus and *Nosema* prevalence. Queen exposure to extreme temperatures during shipment can reduce sperm viability as indicated in one real-world shipping event and follow-up laboratory exposure of queens to temperature extremes. Reduced viability with temperature extremes is believed to be the first report of such an impact on queen health. Taken together this data point to areas of concern with queen quality from the breeder and issues with shipping temperatures but do not preclude problems that queens could encounter in the hive such as pesticide exposure.

Beekeepers in these studies removed queens from colonies that they felt were heading colonies in either good or failing health and these colony assessments are made using an overall gestalt or feeling that the beekeeper gets when looking at the bees and brood within the colony. A major part of the colony assessment is the “brood pattern”, a relative measure of how well the queen has laid eggs and how well those eggs have developed into larvae and pupae. Beekeepers associate poor brood patterns with failing colonies and this is one of the major factors, along with adult bee population, that they used to determine if queens originated from healthy or failing colonies. More precisely, brood pattern can be measured in terms of number of empty cells within a given area of cells contain larvae or pupae that are sealed (e.g. and area of 100 cells might contain 10 empty cells and thus have 10% of the total cells missing). It is assumed that the queen fills all cells in a given area with eggs and that the missing cells are individuals that have died and been removed. Previous research has indicated that when brood patterns contain more than 20% open cells then colonies are more likely to die [4]. It is not certain at this time if queens with less than 50% viable sperm lay poorer brood patterns. The queens surveyed in this research from colonies rated by the beekeeper as being good or failing, strongly suggest a link between poor brood pattern, colony health and low sperm viability.

Commercially available newly-mated queens surveyed showed wide variation in both sperm viability and pathogen levels. In the queens obtained in July and September there was one breeder (#2, Fig 4) that was consistently high at ca. 90% viability. Surprisingly, the breeder with the lowest average viability in July (breeder #4) had the highest viability in September; which points to the numerous factors that might influence viability and that quality is not consistent across breeders and time. Previous surveys of queen health in the U.S. had shown similar levels of DWV and BQCV virus [5] but we report much higher prevalence for *N*. *ceranae*. The actual effects of various viruses on queen health remains unknown (Gauthier et al. 2011). It is known that infection with *Nosema apis* can cause premature supercedures [41]. Thus the high prevalence levels of *Nosema ceranae* reported here are likely of concern. Perhaps of most concern was the low sperm viability measured from several breeders which may indicate potential issues with drone health. Drones have been shown to be sensitive to pesticides used to control mites [16, 17] and to Varroa infestation; all having a negative impact on drone health. Drones could mate with queens and have viable sperm at the time of mating but that sperm may be inferior in quality with pesticide exposure and die in as few as 6 weeks after mating, as noted when drones were reared in the presence of coumaphos [28]. All aspects of producing healthy and fertile drones need to be investigated to reduce the low viability in newly mated queens reported here. Lastly, simple improvements in the queen rearing process may help reduce virus and *Nosema* levels and thus produce healthier queens.

We cannot blame all queen issues on the queen breeders. Many of the queens examined in the current research were of high quality. What is needed is to further identify factors in the drone and queen production process that result the well mated and long-lived queens. Work with the shipping companies to reduce or eliminate extreme temperatures during shipment or find ways to modify the queen shipping containers to allow attendant bees to better heat or cool the queens during shipment. Reducing pesticide exposure to drones and queens at the colony level should improve sperm viability and queen longevity. A clear relationship between low sperm viability and failing colonies are reported here and shipping temperatures is one possible explanation for the observed low sperm viability in queens. Additional research is needed on drone health and exposure to pesticides prior to mating and on the possible role of pesticides in queen health at the colony level.

## Supporting Information

S1 Data Supporting data for Figs 1–5 and Table 1, each Figure or Table is on a separate spreadsheet within the S1 Excel File. (XLSX)
