# Correction: Colony Failure Linked to Low Sperm Viability in Honey Bee (*Apis mellifera*) Queens and an Exploration of Potential Causative Factors

**DOI:** 10.1371/journal.pone.0155833

**Published:** 2016-05-12

**Authors:** Jeffery S. Pettis, Nathan Rice, Katie Joselow, Dennis vanEngelsdorp, Veeranan Chaimanee

The figure legends for Figs 2 and 3 are incorrect and should be reversed. Please see the correct figure legends below.

**Fig 2:**
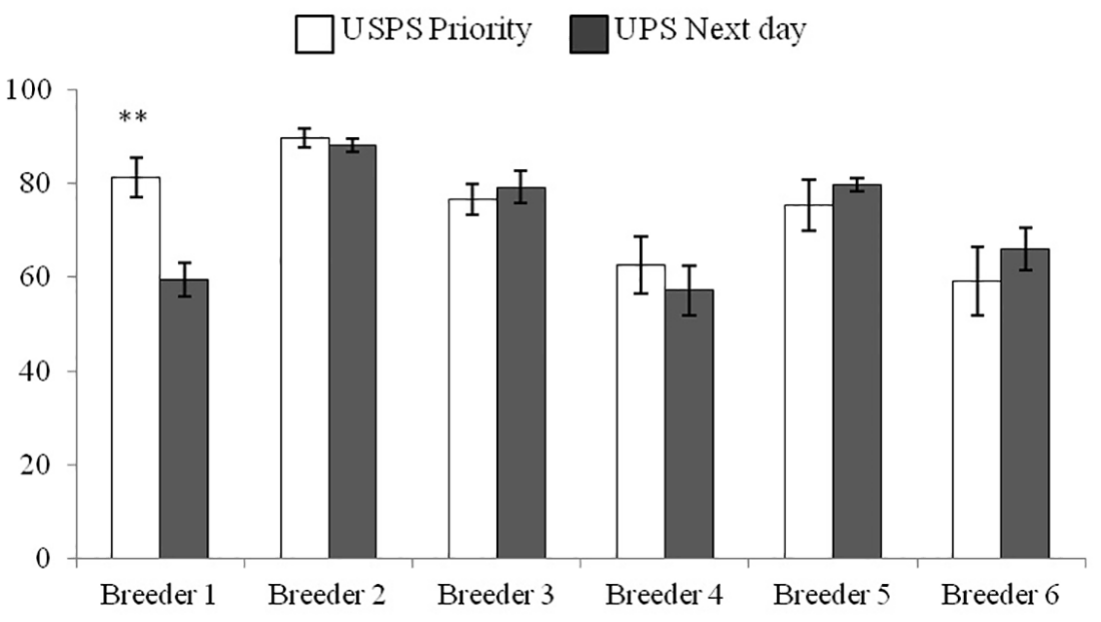
Percent sperm viability in queens (n = 10 per shipping method / breeder) obtained from six queen breeders across the U.S. utilizing two shipping methods, US Postal Service Priority (USPS) and United Parcel Service (UPS). Queen shipments contained temperature monitors and significant difference in viability by shipping method from Breeder #1 represent a cold spike where the queens were exposed to 8°C for two hours. ** indicated significant differences (p<0.01, paired t-test).

**Fig 3:**
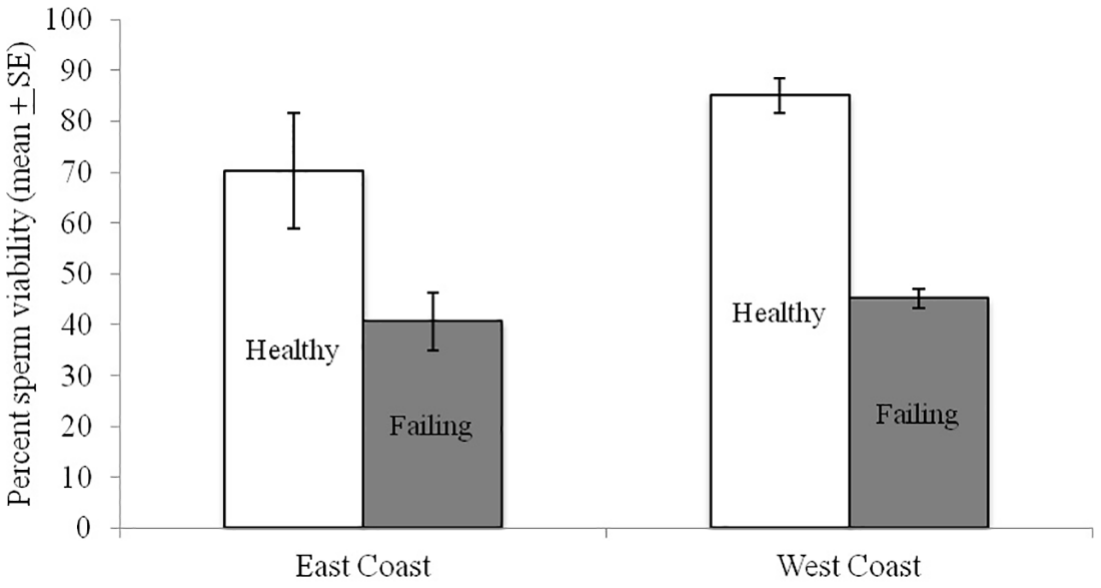
Sperm viability in queens heading colonies that were rated as in healthy or failing health. Queens are from two commercial beekeeping operations, colony heath was rated by the beekeeper and sperm viability assessments conducted blind relative to hive ratings. Data are from queens removed from a single apiary in either the east coast (healthy n = 8 and failing n = 14) or west coast (healthy n = 9 and failing n = 12) and asterisk indicate significant differences in viability within apiaries (paired t-test P<0.05).

## References

[1] Pettis JS, Rice N, Joselow K, vanEngelsdorp D, Chaimanee V (2016) Colony Failure Linked to Low Sperm Viability in Honey Bee (*Apis mellifera*) Queens and an Exploration of Potential Causative Factors. PLoS ONE 11(2): e0147220 doi: 10.1371/journal.pone.0147220 26863438 10.1371/journal.pone.0147220 PMC4749221

